# Effect of Domain Size, Boundary, and Loading Conditions on Mechanical Properties of Amorphous Silica: A Reactive Molecular Dynamics Study

**DOI:** 10.3390/nano10010054

**Published:** 2019-12-25

**Authors:** Truong Vo, Brett Reeder, Angelo Damone, Pania Newell

**Affiliations:** 1 Department of Mechanical Engineering, The University of Utah, UT 84112, USA; truong.vo@utah.edu (T.V.); brettreeder63@hotmail.com (B.R.); 2 Department of Mechanical and Process Engineering, Technical University of Kaiserslautern, 67663 Kaiserslautern, Germany; angelodiscovery89@gmail.com

**Keywords:** nanoscale mechanics, size effect, amorphous silica, reactive molecular dynamics, bimodular materials, anisotropic materials

## Abstract

Mechanical properties are very important when choosing a material for a specific application. They help to determine the range of usefulness of a material, establish the service life, and classify and identify materials. The size effect on mechanical properties has been well established numerically and experimentally. However, the role of the size effect combined with boundary and loading conditions on mechanical properties remains unknown. In this paper, by using molecular dynamics (MD) simulations with the state-of-the-art ReaxFF force field, we study mechanical properties of amorphous silica (e.g., Young’s modulus, Poisson’s ratio) as a function of domain size, full-/semi-periodic boundary condition, and tensile/compressive loading. We found that the domain-size effect on Young’s modulus and Poisson’s ratio is much more significant in semi-periodic domains compared to full-periodic domains. The results, for the first time, revealed the *bimodular* and anisotropic nature of amorphous silica at the atomic level. We also defined a “safe zone” regarding the domain size, where the bulk properties of amorphous silica can be reproducible, while the computational cost and accuracy are in balance.

## 1. Introduction

Mechanical properties are a subset of physical properties that are based on the laws of mechanics dealing with energy and forces, as well as their effects on bodies. Mechanical properties help to determine the range of usefulness of a material and establish the service life that can be expected, but also classify and identify materials. Classical laws of mechanics assume that mechanical properties are independent of sample size. However, numerous experimental and numerical studies have indicated the size effects on mechanical properties at the sub-micron scale as presented in review articles [1,2]. These articles summarize two major mechanisms of the size effect as follows: (i) Plastic deformation, which in macroscopic samples is essentially size independent, however it becomes strongly size dependent and intermittent at microscale and below; (ii) The surface-to-volume ratio starts to increase dramatically when the sample is reduced down to nanoscale. In such a situation, surface effects must be taken into account, including the nature of the chemical bond, equilibrium interatomic distances, coordination number of atoms which are different from inside the bulk. Mechanical properties of materials, regardless of length scale, are almost entirely determined by the bonding network at the atomic level [1]. Therefore, to understand the size effect on mechanical properties, studies at nanoscale are essential for the application to larger scales.

Molecular dynamics (MD) approach is an excellent tool to address the demand for nanoscale studies [3,4]. MD has the ability to provide a higher spatial and temporal resolution of the fracture process compared to a continuum mechanics approach. This is because the interaction and distance between atoms in MD are governed by fundamental theories of chemistry. In addition, MD allows us to investigate and elucidate the microscopic properties that cannot be observed by laboratory experiments [1]. The advantages of MD have been demonstrated as it has been used for many applications, such as nanoscale thermal management [5], nanofluidics [6], nano-machining processes [7], etc.

There have been numerous MD studies on size effect on mechanical properties at nanoscale, including nanowires [8,9,10,11], thin film solids [12,13], etc. The key findings in these articles are that: mechanical properties at nanoscale are strongly affected by sample size and these properties converge to bulk values with increasing size. For instance, Heino et al. found that for relatively large systems (>10${}^{5}$ atoms) the moduli of copper turned out to be independent of the system size [14]. In another study, it was revealed that both strength and toughness of silica glass converge well for domains larger than 3 $\times 10^{5}$ atoms [15]. However, there is still a *knowledge gap* in the size effect associated with different boundary and loading conditions.

We *hypothesize* that boundary and loading conditions affect the domain-size dependency on mechanical properties. Boundary conditions (BCs) include periodicity, while loading conditions (LCs) include tensile and compressive loadings. An investigation of these loading types is important as it was indicated that most materials including ceramics, concrete, and some composite, exhibit different tensile and compressive strains under similar applied load in tension or compression [16,17]. These materials are called *bimodular* materials. They deviate from classical elasticity theory, which assumes that materials have the same elastic properties in tension and compression. However, these characteristics are often neglected due to the complexity of their analysis. Despite the fact that bimodular materials have been studied frequently at the continuum level [16,17], to the best of our knowledge, the bimodularity of a material at nanoscale has not been studied in the literature yet. Therefore, in this study, we are also interested in investigating the bimodularity effect at the atomic level. Furthermore, we investigate the effect of domain-size, boundary and loading conditions on mechanical properties of amorphous silica (a-SiO${}_{2}$) by using MD simulations and the state-of-the-art ReaxFF force field [18].

Amorphous silica is selected in this study for two reasons: (i) a-SiO${}_{2}$ is mainly classified as a brittle material, in which a fracture event at the atomic scale determines its mechanical properties [3], and (ii) the potential application of an understanding of fracture process of a-SiO${}_{2}$ to a wide spectrum of fields [19,20,21]. The results in this study will provide very useful information for studies on material failure. Specifically, domain-size has usually been defined arbitrarily regardless of applied boundary and loading conditions as shown in the literature [22,23,24]. Furthermore, multi-scale modeling has been applied recently to provide efficient and accurate insight into the fracture of materials [25,26,27]. In these studies, material properties are computed by MD, then they are passed to the fracture model at the larger scale. The accuracy of MD information (e.g., mechanical properties, bimodularity, and isotropy) affects the reliability of fracture analysis of such systems. Therefore, a proper simulation of the domain-size at atomic level is critical to obtain accurate information for predicting the overall performance of such multi-scale systems.

The remaining part of the paper is organized as follows. In Section 2, we provide details about the computational approach including fundamental background of reactive molecular dynamics (RMD) simulation, development of a-SiO${}_{2}$ model used in this study, and simulation cases and computations. In Section 3, results and discussion will be presented, followed by Section 4 containing the conclusions and closing remarks.

## 2. Numerical Approach

### 2.1. Reactive Molecular Dynamics Simulation

One of the critical challenges in modeling silicates is the availability of an MD force field that is able to reproduce the mechanical properties under different compositions and chemical environments [28]. Classical MD force fields (e.g., Beest-Kramer-van Staten (BKS) [29], Tersoff [30], Stillinger-Weber (SW) [31], and Pedone [32]) are based on structural data and equations of state or cohesive energies, however, because they are rarely designed for simulating high-strain conditions, it causes deviation in tensile properties [22]. ReaxFF [18] is a reactive bond-order based force field and usually derived by fitting against a training set comprising both quantum mechanical and experimental data. ReaxFF has been shown to be an excellent candidate for high-strain conditions and can better predict the mechanical properties of a-SiO${}_{2}$ better compared with two and three bodies force fields [22].

The total energy of the system modeled by ReaxFF includes several energy terms, such as:

(1) $$E_{\mathrm{total}}=E_{\mathrm{bond}}+E_{\mathrm{over}}+E_{\mathrm{under}}+E_{\mathrm{lp}}+E_{\mathrm{val}}+E_{\mathrm{pen}}+E_{\mathrm{tors}}+E_{\mathrm{conj}}+E_{\mathrm{vdW}}+E_{\mathrm{coul}}.$$

$E_{\mathrm{bond}}$ describes the energy of single, double and triple bonds, $E_{\mathrm{over}}$ and $E_{\mathrm{under}}$ represents the energy terms due to complex interactions that lead a specific atom type to have over and under coordination with other atom types, respectively. $E_{\mathrm{lp}}$ models the lone electron pairs, $E_{\mathrm{val}}$ is related to the valence angle. $E_{\mathrm{pen}}$ takes into account a penalty coming from cumulative double bonds in a particular valency angle. $E_{\mathrm{tors}}$ describes all different torsional configurations, $E_{\mathrm{conj}}$ represents the conjugated double bonds effect. $E_{\mathrm{vdW}}$ is an energy term that takes into account non-bonded interactions and $E_{\mathrm{coul}}$ is the energy contribution due to electrostatic interactions.

In this study, simulations were carried out using LAMMPS code with the USER-REAXC package [33]. All the interaction and reaction parameters in the a-SiO${}_{2}$ model are obtained from literature [34].

### 2.2. Development of a-SiO${}_{2}$

a-SiO${}_{2}$ was created through a melt-and-quench procedure. For this procedure, a simulation time step of 0.5 fs was used. A similar time-step was used for this procedure in other studies [22,35]. First, a simulation domain of 4.95 × 4.95 × 1.53 nm${}^{3}$ of β-cristobalite was generated containing 2400 atoms in a 2:1 O/Si ratio. The β-cristobalite model was annealed by increasing temperature from 300 to 4000 K for 200 ps using an NVT ensemble (i.e., constant number of atoms, volume, and temperature) to create an amorphous structure. An NVT ensemble was used to avoid evaporation of the silica [22]. Then, the system was cooled down to 300 K at a rate of 5 K/ps using an NVT ensemble. Finally, a-SiO${}_{2}$ model was relaxed at 300 K and a pressure of 1 atm for 200 ps by using an NPT ensemble (i.e., constant number of atoms, pressure, and temperature) for releasing all the accumulated tension during the cooling process and adaptation of the system volume. The dimensions of the equilibrated system (4.902×4.902×1.51 nm${}^{3}$) are similar to the initial system.

**Figure 1:**
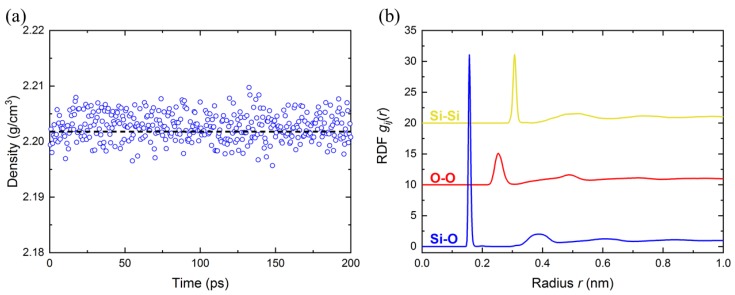
(**a**) Density of a-SiO${}_{2}$ in the relaxation stage (circles). The dashed line denotes the average value (2.202 g/cm${}^{3}$). (**b**) Radial distribution function (RDF) analysis for all pairs of a-SiO${}_{2}$ model. The results of O-O and Si-Si pairs are shifted 10 and 20 up, respectively, for better view.

To verify the a-SiO${}_{2}$ model obtained from our simulation, we show the analysis of our structure in Figure 1. Figure 1a shows density of the a-SiO${}_{2}$ model in the relaxation stage (with an average density of 2.202 g/cm${}^{3}$), which is in an excellent agreement with the experimental measurement (2.2 g/cm${}^{3}$) [36]. Figure 1b shows the radial distribution function (RDF) of all pairs. The data shows a very good agreement in RDF peak positions between MD and experiment [36] as presented in Table 1. Therefore, our a-SiO${}_{2}$ model is verified for further investigations.

### 2.3. Cases Studied and Computations

**Figure 2:**
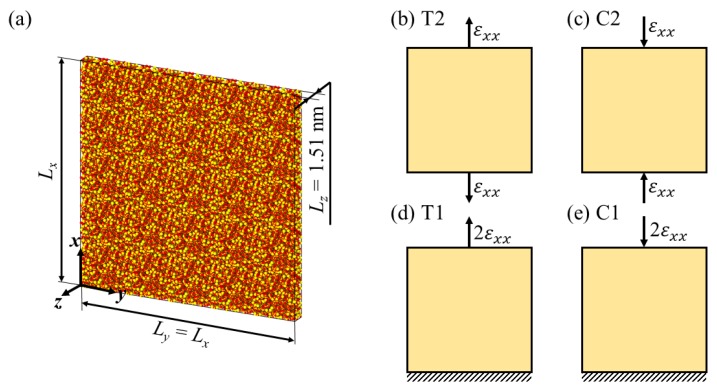
(**a**) Dimensions of a simulation domain of a-SiO${}_{2}$, where $L_{x}=L_{y}$ and $L_{z}=$ 1.51 nm. $L_{x}$ is varied to study the effect of simulation domain. (**b**–**e**) Different simulation sets are employed in this study. Specifically, (**b**,**c**) the simulation domain is fully periodic and subjected to mechanical loading on both sides. T2 and C2 denote tensile and compressive loading on both sides, respectively. (**d**,**e**) Simulation domain is semi-periodic with free surfaces in the *x*-direction, where the bottom surface is fixed and the top surface is subjected to mechanical loading. T1 and C1 denote tensile and compressive loading on one side, respectively.

Figure 2a shows an example of a simulation domain. The unit of a-SiO${}_{2}$ sample obtained previously is replicated in the *x*- and *y*-directions by an identical number of units. Thus, dimensions in these directions are equal ($L_{x}=L_{y}$). These lengths are varied to investigate the effect of simulation domain. In this study, the domain thickness remained constant ($L_{z}=1.51$ nm).

To study the effect of domain-size, boundary and loading conditions, we employ four different simulation sets as follows. Note that we assign each set an ID for convenience.

- T2—simulation domain is fully periodic and subjected to tensile loading on both sides in the *x*-direction, cf. Figure 2b.
- C2—simulation domain is fully periodic and subjected to compressive loading on both sides in the *x*-direction, cf. Figure 2c.
- T1—simulation domain is periodic in the *y*- and *z*-directions, while in the *x*-direction, the tensile loading is applied to the top surface as the bottom surface is fixed, cf. Figure 2d.
- C1—simulation domain is periodic in the *y*- and *z*-directions, while in the *x*-direction, the compressive loading is applied to the top surface as the bottom surface is fixed, cf. Figure 2e.

The simulation sets T2 and C2 are referred to full-periodic cases, while T1 and C1 are referred to semi-periodic cases. In each set, $L_{x}$ and $L_{y}$ are varied from 4.902 to 39.261 nm. Table 2 shows domain dimensions, domain volume, $V_{d}$, and number of atoms, *N*, used in this study. In full-periodic cases (T2 and C2), the simulation domain is linearly expanded with a constant axial displacement rate through scaling the coordinates of all atoms along the *x*-direction at every time step. The engineering strain rate on each side is $\varepsilon_{xx}$. In semi-periodic cases (T1 and C1), a grip with a thickness of 0.75 nm is defined at both top and bottom ends. The bottom grip is fixed, while the top grip is subjected to tensile/compressive deformation with a strain rate of $2\varepsilon_{xx}$. Therefore, the deformation rate in all simulations remain the same regardless of applied boundary and loading conditions.

In all simulations, we set $\varepsilon_{xx}=5\times 10^{9}$ s${}^{-1}$. The strain-rate dependence on modulus of a-SiO${}_{2}$ was studied in the literature [22]. The authors found that as long as the applied strain-rate is smaller than $2.5\times 10^{11}$ s${}^{-1}$, the bulk modulus can be reproduced. By using a Nose-Hoover thermostat/barostat in the NPT ensemble, the temperature and pressure in *y*- and *z*-directions are controlled at 300 K and 1 atm, respectively. The time step is set to 0.25 fs. A similar time step was used in ReaxFF MD simulations on material failure as performed by other researchers [28,35,37].

Engineering stress σ is calculated using the classical definition of virial stress defined as [38]:

(2) $$\sigma =\frac{F}{A_{0}},$$

where *F* is the total inter-atomic force and $A_{0}$ is the initial cross-sectional area. The engineering strain rate ε is calculated as:

(3) $$\varepsilon =\frac{\Delta L}{L_{0}}=\frac{L-L_{0}}{L_{0}},$$

where *L* and $L_{0}$ are deformed and initial sample length, respectively.

## 3. Results and Discussion

The effects of domain-size, boundary and loading conditions are characterized through the global stress-strain curve, Young’s modulus *E*, Poisson’s ratio ν, and bond deformation process. Young’s modulus and Poisson’s ratio are selected among various mechanical properties as they are fundamental and most used properties in characterizing mechanical performance of any materials in elastic region and beyond. To verify the results, we compared the mechanical properties obtained from our simulations with the corresponding ones from the literature as shown in Table 3. Young’s modulus and Poisson’s ratio obtained in all simulations can be found in Table A1 in the Appendix A.

### 3.1. Global Stress-Strain Curve

**Figure 3:**
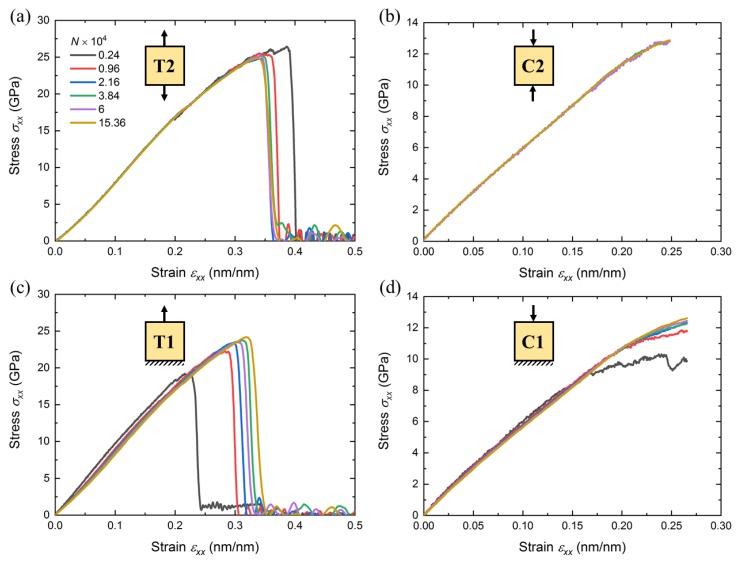
Global stress-strain curves for all scenarios investigated in this study including (**a**) T2, (**b**) C2, (**c**) T1, and (**d**) C1 simulations.

Figure 3 shows the global stress-strain curves that were obtained from all simulations. In tension cases (T2 and T1), the simulations were performed until the a-SiO${}_{2}$ sample was completely broken. In compression cases (C2 and C1), the simulations were stopped when the strain of 0.25 was achieved. From Figure 3, one can observe that there is a negligible difference in terms of domain size in both compression and tension cases subjected to full-periodic boundary conditions (T2 and C2). This is mainly due to the fact that these cases were fully periodic and domains are considered infinity long. On the other hand, the stress-strain curve associated with T1 and C1 were highly influenced by the domain size. This is due to the presence of free surfaces in simulation domain. For the applied boundary conditions in this study, the fracture strength (critical stress) decreases with decreasing domain size in T1 simulations. The influence of periodicity can be described using the arguments the number of atoms as:

(4) $$N=N_{b}+N_{s},$$

where the total number of atoms *N* in a system includes two contributions: the bulk atoms $N_{b}$ and the surface atoms $N_{s}$. For T2 and C2 simulations, Equation (4) reduces to $N=N_{b}$, and the effect of domain-size can therefore be neglected as all the atoms are considered as bulk atoms. However, for T1 and C1 simulations, with decreasing the domain size, the number of atoms in the bulk decreases more rapidly in comparison with the number of surface atoms. This leads to an increase of the surface-to-volume ratio. Under such conditions, the contribution of surface atoms is enhanced with decreasing the domain-size, leading to the rearrangement of interior atoms [46,47]. Therefore, the domain-size impacts the stress-strain curve for both T1 and C1 simulations, with more pronounced effects in cases with a smaller domain size.

### 3.2. Young’s Modulus and Bimodularity

**Figure 4:**
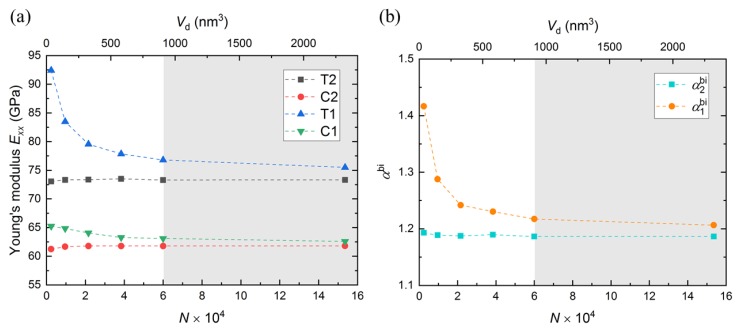
(**a**) Young’s modulus in the *x*-direction, $E_{xx}$, and (**b**) bimodular factor $\alpha^{\mathrm{bi}}$ from all simulations. The domain-size effect is considered by both number of atoms in the domain *N* and simulation domain volume $V_{d}$. $\alpha_{2}^{\mathrm{bi}}$ and $\alpha_{1}^{\mathrm{bi}}$ describe the bimodularity of T2 and C2, and of T1 and C1 simulations, respectively. The gray shaded area describes the "safe zone", which is discussed in Section 3.5.

Young’s modulus in the *x*-direction, $E_{xx}$, is determined as the slope of the global stress-strain curve ($E_{xx}$-$\varepsilon_{xx}$) up to 5% strain through linear regression. Figure 4a shows the Young’s modulus obtained from all simulations. At first glance, one can conclude that the data obtained from the simulations is in good agreement with the experimental data and other ReaxFF simulation data (Table 3). Young’s modulus from tensile simulations (T2 and T1) are higher that the corresponding ones from compressive simulations (C2 and C1). Figure 4a also reveals that with decreasing the domain size, the Young’s modulus deviates from the Young’s modulus of the larger domains. This deviation depends on applied boundary and loading conditions. The deviation in Young’s modulus between the smallest and biggest domains is 22.4% for T1 case, 4.3% for C1 case, and less than 1% for T2 and C2 cases.

Wang and Li proposed the following surface stress concept to predict the Young’s modulus [2,48]:

(5) $$E=E_{b}+\sigma_{s}\left(1-\nu \right)\frac{a}{L},$$

where *E* and $E_{\mathrm{bulk}}$ are the Young’s modulus of a system and of a bulk region, respectively. $\sigma_{s}$ is surface stress, *a* is a geometry parameter, and *L* is the domain size in the loading direction. If the domain is extremely large (L→∞), the contribution of surface stress is neglected and $E\to E_{bulk}$. which confirms the results for full-periodic cases in both tension and compression. On the other hand, a decrease in the domain size indicates an increase in the surface-to-volume ratio and, thus higher contributions from surface stress (Equation (5)). This leads to an increase in Young’s modulus, which explains the higher variation in Young’s modulus in semi-periodic cases (T1 and C1).

As mentioned earlier, the theory of linear elasticity assumes that materials have the same elastic properties in tension and compression, which is a simplified interpretation [16]. However, Figure 4a shows clear distinction between Young’s moduli in tension ($E_{xx}^{t}$) and compression ($E_{xx}^{c}$), indicating a-SiO${}_{2}$ behaves as a *bimodular* material at the atomic level if we define the bimodular factor $\alpha^{\mathrm{bi}}$ as:

(6) $$\alpha^{\mathrm{bi}}=\frac{E_{xx}^{t}}{E_{xx}^{c}}.$$

Figure 4b shows the bimodularity as a function of the domain sizes, boundary and loading conditions. The bimodularity in all simulations is larger than 1, which means a-SiO${}_{2}$ is stiffer in tension than compression. As bimodularity is a product of Young’s modulus, it also shows similar dependence on domain-size, boundary and loading conditions. Figure 4b shows that the bimodularity of a full-periodic domain ($\alpha_{2}^{\mathrm{bi}}$) is not affected by domain-size. However, in simulations with free surfaces ($\alpha_{1}^{\mathrm{bi}}$), bimodularity increases as the domain-size becomes smaller. The high relative difference of 14.8% from the smallest to the largest domain is another indicator of the importance of the simulation domain size on material properties. Considering the results of large domains, The bimodularity factor converges to the value of 1.2 for all cases if the domain size is sufficiently large.

### 3.3. Poisson’s Ratio and Isotropy

**Figure 5:**
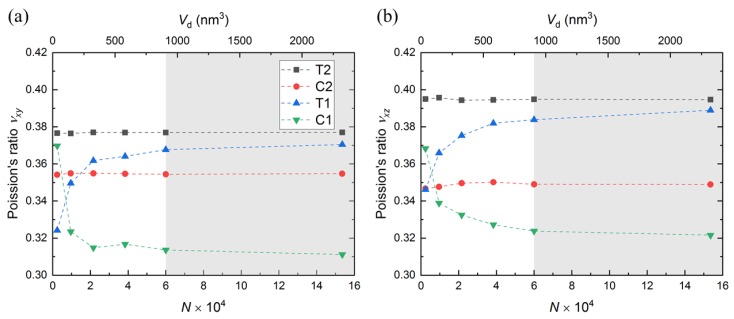
Poisson’s ratio (**a**) $\nu_{xy}$ and (**b**) $\nu_{xy}$ obtained from all simulations. The domain-size effect is considered by both number of atoms in the domain *N* and simulation domain volume $V_{d}$. The gray shaded area describes the “safe zone”, which is discussed in Section 3.5.

Poisson’s ratio, ν, is calculated as: $\nu =-\varepsilon_{\mathrm{lateral}}/\varepsilon_{\mathrm{axial}}$, where $\varepsilon_{\mathrm{lateral}}$ and $\varepsilon_{\mathrm{axial}}$ are lateral and axial strains, respectively. Accounting for both the lateral *y*- and *z*-directions in the calculation of Poisson’s ratio leads to $\nu_{xy}$ and $\nu_{xz}$ as shown in Figure 5a,b, respectively. Our data matches well with other simulation data reported in the literature as shown in Table 3.

It can be seen from Figure 5 that the Poisson’s ratio follows similar trends as the Young’s modulus. In another words, there is a negligible difference in full-periodic simulations (T2 and C2), while there is a distinct difference in semi-periodic simulations (T1 and C1) as the domain size changes. As strain in x direction (e.g., $\varepsilon_{xx}$) remained the same at every time step for all simulations, a larger Poisson’s ratio implies a larger strains in y and z directions (e.g., $\varepsilon_{yy}$ or $\varepsilon_{zz}$), which indicates that a-SiO${}_{2}$ contracts easier than it expands. This Poisson’s ratio mismatch is likely due to the porous structure of a-SiO${}_{2}$, where there are many sub-nanometer pores inside.

Although a-SiO${}_{2}$ has been classified as an isotropic material at the macroscale [49], Figure 5 shows that Poisson’s ratio in xy direction (e.g., $\nu_{xy}$) is slightly different than Possion’s ration in xz direction (e.g., $\nu_{xz}$). Isotropic material behavior plays an important role in modeling material failure at larger scales. However, most models assume an isotropic material behavior, which may not be a valid assumption for micro-/nano-structures [50,51].

We define the isotropic factor $\alpha^{\mathrm{iso}}$ as:

(7) $$\alpha^{\mathrm{iso}}=\frac{\nu_{xy}}{\nu_{xz}}.$$

**Figure 6:**
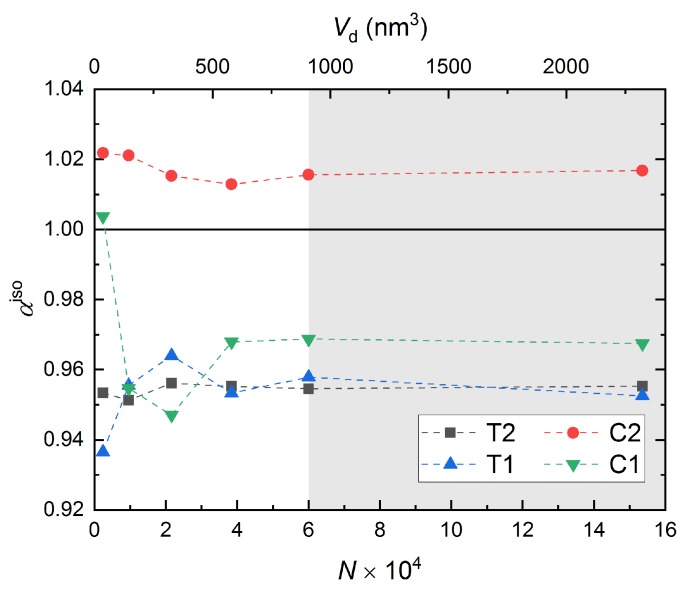
Characterization of isotropy of a-SiO${}_{2}$ through the parameter $\alpha^{\mathrm{iso}}$ from all simulations. The black solid line indicates $\alpha^{\mathrm{iso}}=1$. The gray shaded area describes the “safe zone”, which is discussed in Section 3.5.

If $\alpha^{\mathrm{iso}}=1$, the material is isotropic; otherwise, the material is considered as anisotropic. The isotropic factor obtained from all simulations is shown in Figure 6. All cases manifest slightly anisotropic behavior at atomic level regardless of domain-size, and applied boundary and loading conditions. Interestingly, the full-periodic simulation under compression reveals that the material deforms more in the *x*-*y* plane compared with the *x*-*z* plane, while this behavior is reversed in other cases. This behavior might be due to the collapse of nanopores under uniaxial compression from both sides.

The anisotropy of a-SiO${}_{2}$ is attributed to the involvement of ductility during deformation as revealed by several studies [52,53]. The contribution of ductility in a-SiO${}_{2}$ at nanoscale is caused by a portion of energy from the stored strain energy that is converted into heat or unrecoverable inelastic deformation [28]. Note that, to simplify the analysis and to be similar to other RMD studies [15,28,35,44], plane stress conditions were used in the *x*-*y* plane by setting $L_{z}\ll L_{x}$ and $L_{z}\ll L_{y}$ for this study. An accurate analysis, as well as a comparison in anisotropy of a-SiO${}_{2}$ under different boundary and loading conditions will be a part of our future work, where the dimensions in all the directions remain the same.

### 3.4. Distribution of Si-O Bond

**Figure 7:**
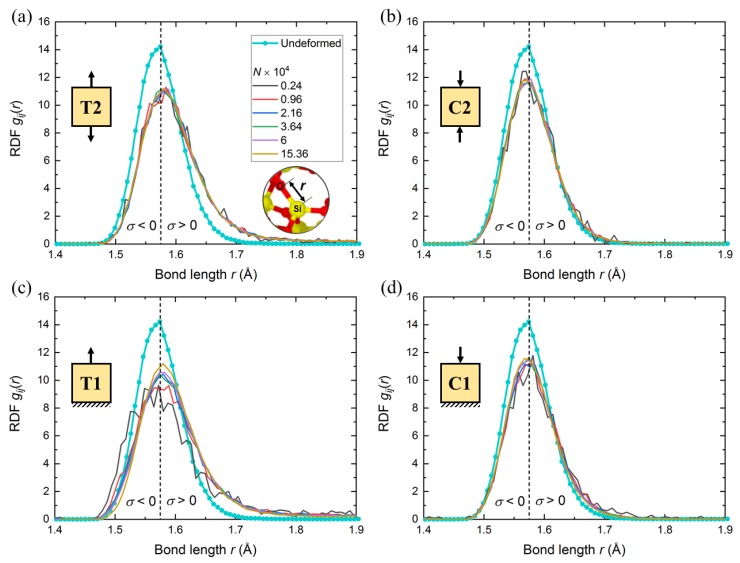
Radial distribution function RDF $g_{ij}\left(r\right)$ of Si-O bond distribution under deformation (solid lines) compared to undeformed state (solid line with solid circles). All the data of undeformed state are obtained at 20% strain of (**a**) T2, (**b**) C2, (**c**) T1, and (**d**) C1 simulations. The compressive and tensile regions are described by σ<0 and σ>0, respectively. An inset in (**a**) describes the Si-O bond length *r*.

Figure 7 shows the RDF of Si-O bond under deformation (including stretching, σ>0, and contraction, σ<0, in length) at 20% strain. The results are compared with the bond distribution at an undeformed state. When a-SiO${}_{2}$ sample is not deformed, the distribution is nearly symmetric with a minimum, maximum, and average bond length of 1.478, 1.718, and 1.581 *Å*, respectively.

Figure 7 illustrates the variations in bond distribution compared to the undeformed state. As it can be clearly seen, the bond distributions in tensile simulations were shifted to the right, however, such a variation in compressive simulations is very small. This observation indicates that the volumetric change is higher in tensile simulations, leading to a higher Poisson’s ratio in tensile cases. For semi-periodic cases (T1 and C1), there is a small domain-size effect on the Si-O bond distribution due to the impact of the fixed bottom layer. For T1 simulations, minimum bond length is lower in smaller domains compared to the larger ones, which leads to less volumetric changes in the smaller domains. As a result, Poisson’s ratio increases with a decreasing domain size in T1 simulations. In contrast, maximum bond length is higher in smaller domains compared to the larger ones in C1 simulations, which causes a higher volumetric change in the smaller domains. Consequently, Poisson’s ratio increases with increasing domain-size in C1 simulations.

### 3.5. Computational Cost and Accuracy

The cost in the use of the high-performance computers is specified in the so-called utilization units (UU) as:

(8) UU=(NumberofCPUcores)×hours/1000.

For example, a job that occupies 16 CPU cores for a day consumes 16×24/1000=0.384 UU. In our simulations, each core was an INTEL Xeon Gold 6130 scalable processor with a clock speed of 2.1 GHz and 192 GB of total memory. The computation cost for each simulation is shown in Table 4.

As multiscale materials modeling becomes increasingly important in many applications, it is essential to check the accuracy of the results at each scale. Errors across multiscale modeling from atomistic level may be caused by MD input parameters (force field parameters), as well as input parameters at larger scales [54]. In this study, we assumed that there is no error associated with MD as ReaxFF is a reactive bond-order based force field and it can reproduce mechanical properties very well. Thus, the “safe zone” can be used as a domain in which both accuracy and computational cost are optimized. Furthermore, the concept of representative volume element (RVE) has been extensively used in the multiscale modeling [55,56], and it has also been applied to MD studies recently [57]. The choice of RVE that can accurately capture the material’s bulk-scale mechanical behavior is critical. Therefore, even though the proposed “safe zone” is valid just for the scenarios investigated in this paper, it suggests how to choose RVE size at nanoscale.

**Figure 8:**
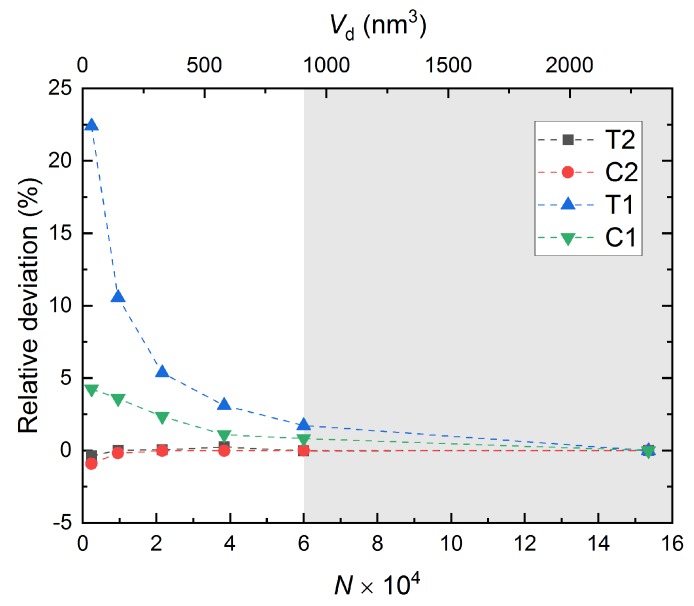
The relative deviation in Young’s modulus from each simulation to the largest simulation. The gray shaded area describes the “safe zone” where the errors are reasonably small.

As the effect of domain size on Young’s modulus and Poisson’s ratio is similar, the results of Young’s modulus are used in driving the discussion on accuracy in this section. Assuming the result from the largest domain is an exact solution, Figure 8 shows the relative deviation of Young’s modulus for each domain with respect to corresponding modulus obtained from the largest domain. As expected, the deviation in full-periodic simulations (T2 and C2) is almost zero. However, for semi-periodic simulations (T1 and C1), the error increases as the domain size becomes smaller. We observed that all the errors lay within 2% from the second largest domains ($N\geq 6\times 10^{4}$ or $V_{b}\geq 907,118$ nm${}^{3}$). Therefore, for our scenarios, we propose a “safe zone” in which the simulation domain effect is negligible. However, similar case studies can be performed for other cases, as well.

## 4. Conclusions

In this paper, reactive molecular dynamics simulations were used to investigate the effect of domain size, as well as the loading and boundary conditions, on mechanical properties of amorphous silica. Various scenarios based on tensile/compressive loading, full-/semi-periodic boundary condition, as well as different domain sizes, were numerically explored. The a-SiO${}_{2}$ model was verified by comparing the density and RDF analysis with experimental measurements from the literature [36]. The number of atoms in a simulation domain was systematically increased from 0.24 $\times \;10^{4}$ to 15.36 $\times 10^{4}$. Young’s modulus and Poisson’s ratio were obtained from the simulations. Our results indicated that:

- Mechanical properties converge with increasing domain size.
- With the presence of free surfaces in semi-periodic cases, the impact of domain size is much more significant than full-periodic cases.
- Amorphous silica exhibits strong *bimodular* behavior and slight anisotropy at the atomic level. Young’s modulus in tension is higher than in compression, while Poisson’s ratio in *x*-*y* plane and *x*-*z* plane are slightly different from each other.
- A “safe zone” defined as a zone where accuracy and computational cost are balanced. Defining such a zone is necessary for multiscale models, as well as defining RVE at nanoscale. In this zone, bulk properties can be reproduced with good accuracy.

The bimodular and anisotropic characteristics of a-SiO${}_{2}$ at the atomic level are very important for multiscale models as classical theory are usually applied, which assumes materials are isotropic and have the same elastic properties in tension and compression. A deeper analysis on anisotropy of a-SiO${}_{2}$ at the atomic level will be a part of our future work.

## Figures and Tables

**Table 1:** RDF first and second peak positions of all pairs in a-SiO${}_{2}$. The data includes both the results from our simulation and experiment from literature [36], which are in good agreements.

Structural Parameters	Our Simulation Results	Experimental Results [36]
Si-Si RDF 1st peak position (nm)	0.3071	0.3077
Si-Si RDF 2nd peak position (nm)	0.5193	0.5182
O-O RDF 1st peak position (nm)	0.2538	0.2626
O-O RDF 2nd peak position (nm)	0.4896	0.5097
Si-O RDF 1st peak position (nm)	0.1633	0.1608
Si-O RDF 2nd peak position (nm)	0.3969	0.4061

**Table 2:** Number of atoms *N* and simulation domain volume $V_{d}$ corresponds to every simulation domain-size studied. The initial simulation box thickness is always a constant, $L_{z}=1.51$ nm.

$L_{x}=L_{y}$ (nm)	$V_{d}$ (nm${}^{3}$)	$N\times 10^{4}$
4.902	36.285	0.24
9.804	145.139	0.96
14.706	326.562	2.16
19.608	580.555	3.84
24.510	907.118	6.00
39.216	2322.221	15.36

**Table 3:** Example of Young’s modulus, *E*, and Poisson’s ratio, ν, of a-SiO${}_{2}$ from experiment and simulational studies reported in literature. The bound refers to the maximum and minimum values of reported experimental and numerical data.

	Reference Study	*E* (GPa)	ν
**Experiment**	Freund and Suresh [39]	80	0.22
	Deschamps et al. [40]	71.5	0.176
	Wiederhorn [41]	72.1	...
	Wallenberger et al. [42]	69	...
	Bansal and Doremus [43]	72.9	...
Bound		69–80	0.176–0.22
**ReaxFF simulation**	Hao and Hossain [15]	69.1	0.25–0.32
	Chowdhury et al. [22] ${}^{a}$	75.4–76.68	...
	Chowdhury et al. [44]	74	0.39
	Rimsza et al. [28]	...	0.31
	Yu et al. [45]	80.4 ± 1.9	...
	Mei et al. [35]	60	0.296
Bound		60–82.3	0.25–0.39

${}^{a}$ Values for strain-rate smaller than $2.5\times 10^{11}$ s${}^{-1}$.

**Table 4:** Computational cost in each simulation is shown in utilization units (UU).

Simulation Set	*N* $\times \;10^{4}$	UU	Simulation Set	*N* $\times \;10^{4}$	UU
T2	0.24	0.164	T1	0.24	0.411
	0.96	0.323		0.96	0.420
	2.16	0.575		2.16	0.804
	3.84	1.089		3.84	1.105
	6.00	1.553		6.00	1.704
	15.36	3.107		15.36	3.129
C2	0.24	0.140	C1	0.24	0.140
	0.96	0.241		0.96	0.241
	2.16	0.368		2.16	0.365
	3.84	0.539		3.84	0.532
	6.00	0.745		6.00	0.740
	15.36	1.561		15.36	1.565

## Abbreviations

The following abbreviations are used in this manuscript:

MD	Molecular dynamics
RMD	Reactive molecular dynamics
BCs	Boundary conditions
LCs	Loading conditions
RDF	Radial distribution function

## Appendix A. Simulation Data

The results of all the simulations carried out in the paper are presented in Table A1, including Young’s modulus, $E_{xx}$, and Poisson’s ratio, $\nu_{xy}$ and $\nu_{xz}$.

**Table A1:** Mechanical properties obtained from all simulations in this study.

Simulation Set	N $\times 10^{4}$	$E_{\mathrm{xx}}$ (GPa)	$\nu_{\mathrm{xy}}$	$\nu_{\mathrm{xz}}$
T2	0.24	73.052	0.377	0.395
	0.96	73.319	0.376	0.396
	2.16	73.359	0.377	0.394
	3.84	73.500	0.377	0.395
	6.00	73.300	0.377	0.395
	15.36	73.318	0.377	0.395
C2	0.24	61.231	0.350	0.347
	0.96	61.677	0.355	0.348
	2.16	61.782	0.355	0.350
	3.84	61.779	0.355	0.350
	6.00	61.786	0.354	0.349
	15.36	61.791	0.355	0.349
T1	0.24	92.412	0.324	0.346
	0.96	83.484	0.350	0.366
	2.16	79.549	0.362	0.375
	3.84	77.850	0.364	0.382
	6.00	76.802	0.368	0.384
	15.36	75.500	0.370	0.389
C1	0.24	65.246	0.370	0.368
	0.96	64.832	0.323	0.339
	2.16	64.056	0.315	0.332
	3.84	63.270	0.317	0.327
	6.00	63.092	0.314	0.324
	15.36	62.581	0.311	0.322
